# Supplementary material for: Rapid learning and unlearning of predicted sensory delays in self-generated touch
Source: eLife. 2019 Nov 18;8:e42888. doi: 10.7554/eLife.42888 (PMC6860990; doi:10.7554/eLife.42888)
Supplement: Figure 2—source data 1. [file elife-42888-fig2-data1.docx]

**Fig. 2, Source Data 1.** Mean PSE (± s.e.m.) for each condition.

| **Condition** | **PSE** |
| --- | --- |
| [0 ms, 0 ms] | 1.831512 ± 0.02500018 |
| [0 ms, 100 ms] | 1.978940 ± 0.02061445 |
| [100 ms, 0 ms] | 1.904739 ± 0.02703384 |
| [100 ms, 100 ms] | 1.891190 ± 0.02870765 |
| baseline | 1.966662 ± 0.02627352 |
