# Supplementary material for: Rapid learning and unlearning of predicted sensory delays in self-generated touch
Source: eLife. 2019 Nov 18;8:e42888. doi: 10.7554/eLife.42888 (PMC6860990; doi:10.7554/eLife.42888)
Supplement: Figure 2—source data 2. [file elife-42888-fig2-data2.docx]

**Fig. 2, Source Data 2.** Attenuation shifts in immediate touch (unlearning) and delayed touch (learning).

| **ID** | **Unlearning** | **Learning** |
| --- | --- | --- |
| 1 | -0.224883480765484 | 0.00995905411082409 |
| 2 | -0.0976652959898634 | 0.204162032162774 |
| 3 | -0.0101709883059042 | 0.122774795164894 |
| 4 | 0.0487955255706123 | -0.0420199825834895 |
| 5 | -0.0557652734435286 | 0.204893051002174 |
| 6 | 0.204101532758413 | -0.0810187076305466 |
| 7 | 0.18529364196365 | 0.208561689974247 |
| 8 | 0.145520879246424 | 0.0606485640863339 |
| 9 | 0.164216851096042 | 0.175003533152346 |
| 10 | 0.0604848337234947 | 0.0574211340684754 |
| 11 | -0.00257159934637174 | -0.0976611524096009 |
| 12 | 0.145061314298614 | 0.202507658099889 |
| 13 | -0.0721777089745321 | -0.18794870329754 |
| 14 | 0.0259657791650905 | 0.0171807915288031 |
| 15 | -0.109946528459649 | -0.0834956742649338 |
| 16 | 0.025813667309464 | 0.309725824159139 |
| 17 | 0.130202921556366 | 0.0759499364812497 |
| 18 | -0.0864975348389581 | -0.0334245055419324 |
| 19 | 0.022844263348516 | 0.266180709345256 |
| 20 | -0.0459592770119626 | -0.0290567474303882 |
| 21 | 0.113377273803137 | -0.164159483665889 |
| 22 | 0.11081869058516 | 0.19114120578602 |
| 23 | 0.00505465701974672 | 0.0687584240019559 |
| 24 | -0.107250637384679 | -0.0657090670562901 |
| 25 | 0.202387182086397 | 0.225715434660874 |
| 26 | 0.347702940454679 | 0.325120517074256 |
| 27 | 0.221923054019236 | 0.282554999082602 |
| 28 | 0.445271461170566 | 0.267514434924871 |
| 29 | 0.198988484760573 | 0.100488503435645 |
| 30 | 0.205874375314683 | 0.0407212688529737 |
