# Supplementary material for: Rapid learning and unlearning of predicted sensory delays in self-generated touch
Source: eLife. 2019 Nov 18;8:e42888. doi: 10.7554/eLife.42888 (PMC6860990; doi:10.7554/eLife.42888)
Supplement: Figure 2—source data 3. [file elife-42888-fig2-data3.docx]

**Fig. 2, Source Data 3.** Model parameters for the group fits.

| **Condition** | **Alpha** | **Beta** |
| --- | --- | --- |
| [0 ms, 0 ms] | -11.95208 | 6.52580 |
| [100 ms, 0 ms] | -11.75708 | 6.17254 |
| [0 ms, 100 ms] | -11.45110 | 5.78648 |
| [100 ms, 100 ms] | -11.69868 | 6.18588 |
