# Supplementary material for: Rapid learning and unlearning of predicted sensory delays in self-generated touch
Source: eLife. 2019 Nov 18;8:e42888. doi: 10.7554/eLife.42888 (PMC6860990; doi:10.7554/eLife.42888)
Supplement: Figure 2—source data 4. [file elife-42888-fig2-data4.docx]

**Fig. 2, Source Data 4.** Mean PSE (± s.e.m.) as a function of exposure trials

| **Condition** | **Trials** | **PSE** |
| --- | --- | --- |
| [100 ms, 0 ms] | 0 | 1.797962 ± 0.04301662 |
| [100 ms, 0 ms] | 50 | 1.857475 ± 0.04380556 |
| [100 ms, 0 ms] | 200 | 1.887239 ± 0.02392791 |
| [100 ms, 0 ms] | 500 | 1.910641 ± 0.01798157 |
| [100 ms, 100 ms] | 0 | 1.921397 ± 0.03353579 |
| [100 ms, 100 ms] | 50 | 1.855474 ± 0.03170421 |
| [100 ms, 100 ms] | 200 | 1.830529 ± 0.03266655 |
| [100 ms, 100 ms] | 500 | 1.829101 ± 0.03672500 |
