# Supplementary material for: Rapid learning and unlearning of predicted sensory delays in self-generated touch
Source: eLife. 2019 Nov 18;8:e42888. doi: 10.7554/eLife.42888 (PMC6860990; doi:10.7554/eLife.42888)
Supplement: Figure 3—source data 1. [file elife-42888-fig3-data1.docx]

**Fig. 3, Source Data 1.** Median and interquartile range (IQR) for the frequency of a 150 ms delayed touch being perceived as more ticklish than a 0 ms touch, per condition.

| **Condition** | **Median** | **IQR** |
| --- | --- | --- |
| 0 ms | 0.8 | 0.3 |
| 150 ms | 0.6 | 0.3 |
